# In Situ Investigation of Tensile Response for Inconel 718 Micro-Architected Materials Fabricated by Selective Laser Melting

**DOI:** 10.3390/ma17174433

**Published:** 2024-09-09

**Authors:** Ioannis Filippos Kyriakidis, Nikolaos Kladovasilakis, Eleftheria Maria Pechlivani, Apostolos Korlos, Constantine David, Konstantinos Tsongas

**Affiliations:** 1 Advanced Materials and Manufacturing Technologies Laboratory, Department of Industrial Engineering and Management, School of Engineering, International Hellenic University, 57001 Thessaloniki, Greece; giankyri@iem.ihu.gr (I.F.K.); apkorlos@ihu.gr (A.K.); 2 Centre for Research and Technology Hellas, Information Technologies Institute (CERTH/ITI), 57001 Thessaloniki, Greece; nikoklad@iti.gr (N.K.); riapechl@iti.gr (E.M.P.); 3 Manufacturing Technology and Production Systems Laboratory, Department of Mechanical Engineering, School of Engineering, International Hellenic University, 62124 Serres, Greece; david@ihu.gr

**Keywords:** additive manufacturing, selective laser melting, architected materials, micro-tensile testing, in situ scanning electron microscopy (SEM), finite element analysis

## Abstract

Topology optimization enables the design of advanced architected materials with tailored mechanical properties and optimal material distribution. This method can result in the production of parts with uniform mechanical properties, reducing anisotropy effects and addressing a critical challenge in metal additive manufacturing (AM). The current study aims to examine the micro-tensile response of Inconel 718 architected materials utilizing the Selective Laser Melting Technique. In this context, three novel architected materials, i.e., Octet, Schwarz Diamond (SD), and hybrid Schwarz Diamond and Face Centered Cubic (FCC), were tested in three different relative densities. The specimens were then subjected to uniaxial quasi-static tensile tests to determine their key mechanical properties, including elastic modulus, yield strength, and ultimate tensile strength (UTS), as well as the scaling laws describing the tensile response of each architected material. In situ Scanning Electron Microscopy (SEM) has been performed to observe the structure and grain morphology of the 3D printed specimens along with the phase transitions (elastic, plastic), the crack propagation, and the overall failure mechanisms. The results highlight the effect of the lattice type and the relative density on the mechanical properties of architected materials. Topologically optimized structures presented a 70–80% reduction in overall strength, while the SD and SD&FCC structures presented higher stretching dominated behavior, which was also verified by the *n*-value range (1–2) extracted from the identification of the scaling laws.

## 1. Introduction

The demand for metals with tailored mechanical properties and the plurality of applications has raised the urge to study architected materials that satisfy specific performance criteria [1,2]. Additive manufacturing (AM) is a rising technique with the main advantages being the ability to accurately produce complex geometries, the convenience and flexibility in customization, and the low cost and waste minimization during the fabrication process. It utilizes a wide range of materials from thermoplastics to certain types of metals such as steel, nickel, and titanium alloys [3,4,5,6]. Nickel superalloys especially, have been very popular in the industry due to their exceptional properties [7,8]. An indicative example is Inconel 718, a nickel-based superalloy with excellent strength, wear and fatigue resistance, and heat and corrosion resistance, even at high temperatures, making it a reliable material for use in the aerospace and automotive industries for high-temperature applications [9,10,11]. Inconel 718 is an ideal candidate for additive manufacturing processes due to its ability to maintain structural integrity even at high temperatures [12,13]. Metals are mainly utilized for Powder Bed Fusion (PBF) such as Selective Laser Melting (SLM) and Direct Energy Deposition additive manufacturing. The SLM technique utilizes a high-power laser to completely melt the metallic powders particles in a pattern design as given by the user. The materials used are pre-spread on a substrate surface using a re-coater before laser melting with layer-by-layer distribution. It is the go-to technique for the efficient utilization of steel and nickel alloys, as it allows for the construction of complex structures with excellent accuracy in the internal geometry of the resulting architected 3D printed part. Also, because the powder particles are fully melted and resolidified, more unified and durable structures are being constructed with this technique with properties closer to the theoretical material properties that are similar to other conventional techniques (i.e., molding) [14,15]. 

The versatility in customization and the detailed accuracy of the extracted product in additive manufacturing processes allowed for the introduction of topological optimizations and geometry modifications [16,17]. Topology optimization is used to produce efficient lightweight materials by focusing on the optimization of the unit cell shape, size distribution, and key parameters (e.g., strut dimensions) [18,19]. Optimizing weight distribution allows for the construction of efficient materials with a high strength-to-weight ratio. Depending on the relative density (r.d.) on a specific area, a division into three different categories of metals can be made: foams (r.d. < 10%), lattices (10–50%), and solids (>60%). Lattice structures are 3-dimensional structures that contain a periodic repetition of a pattern called a unit cell. This periodic pattern can be either in two dimensions like the honeycomb structure or in three dimensions (3D). Further, 3D cellular materials are divided into strut-based and sheet-based group, like triply periodic minimal surface (TPMS) structures [1,20,21,22]. Among TPMS structures, the diamond shape has demonstrated a superior mechanical response and high levels of energy absorption, as shown by previously conducted three-point bending tests and finite element analysis (FEA) [16]. In addition to this, hybrid structures constructed have shown better mechanical response than the conventional regular cellular materials, with the Schwarz Diamond (SD) (TPMS diamond) and FCC (Face Centered Cubic) hybrid showing the most promising potential for future applications in products that require specific tensile loads with prime examples being the manufacturing of turbines in the aerospace industry and hydrogen tanks [16,23,24]. In general, the customization of the unit cell structure is a key factor in the construction of architected materials with tailored mechanical properties. 

In this study, three innovative lattice structures were developed with topology optimization software to create 3D designed dog-bone-shaped specimens. The first structure was the strut-based Octet structure, the second type was the sheet-based TPMS diamond structure or else Schwarz Diamond (SD), and the third one was a hybrid Schwarz Diamond and Face Centered Cubic (FCC) structure. Three different relative densities were tested for each type of the above structures. The designed specimens were manufactured utilizing the SLM manufacturing technique. Uniaxial quasi-static tensile tests to evaluate crucial mechanical properties (elastic modulus, yield strength, and ultimate tensile strength (UTS)) were conducted, along with the corresponding scaling laws that describe the tensile response of each examined architected material. Assessing the micro-tensile properties of a structure led to a better correlation between the shape and size of a unit cell and its influence on the mechanical performance of the architected material. Also, it was possible to study the tensile deformation and fracture mechanisms, in the microstructure, in contrast to the conventional macroscopic tensile testing [25,26].

Microscopy methods, both optical and electronic (SEM), have been incorporated into the process of mechanical testing in recent decades [27]. In situ monitoring allows for the understanding of mechanical parameters at the micro-scale such as structure and grain morphology and the deformation mechanisms [28]. This allows the connection to properties on the unit cell and the correlation of them with the bulk mechanical properties [29,30]. Tensile deformation mechanisms have been studied at high temperatures with ex situ tensile testing, but this does not allow for the observation of fracture mechanisms in the unit cell, making the final results estimated and inaccurate [31,32]. 

**Figure 1:**
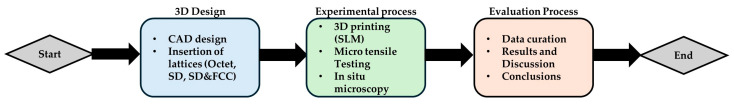
Flow chart of the current work.

The objective of this paper is to conduct a thorough investigation to evaluate the micro-tensile mechanical properties of advanced architected materials made from Inconel 718, along with determining the related scaling laws and the assessment of the relation between the structure and grain morphology to the strain hardening and fracture mechanisms of each material. The novelty of this paper lies in the identification of the micro-tensile response of the specimens and their correlated scaling laws; the observation of the deformation mechanisms; and the correlation to structure, size, and grain morphology of the unit cell in each type of architected material. The results of this paper provide a deeper and more detailed perspective on the overall tensile behavior of Inconel 718 lattice structures, improving the general knowledge on materials with tailored mechanical properties and allowing their introduction in real-life applications. In Figure 1, a brief flow chart of this study is illustrated. In Section 2, a description of the feedstock’s characteristics, the printing process along with the digital and physical equipment for the design process and the equipment used for the mechanical evaluation of the fabricated specimens is presented. In Section 3, the results of the mechanical evaluation process are provided and discussed along with some brief conclusions.

## 2. Materials and Methods

### 2.1. The 3D Design of the Lattice Structure Specimens

**Figure 2:**
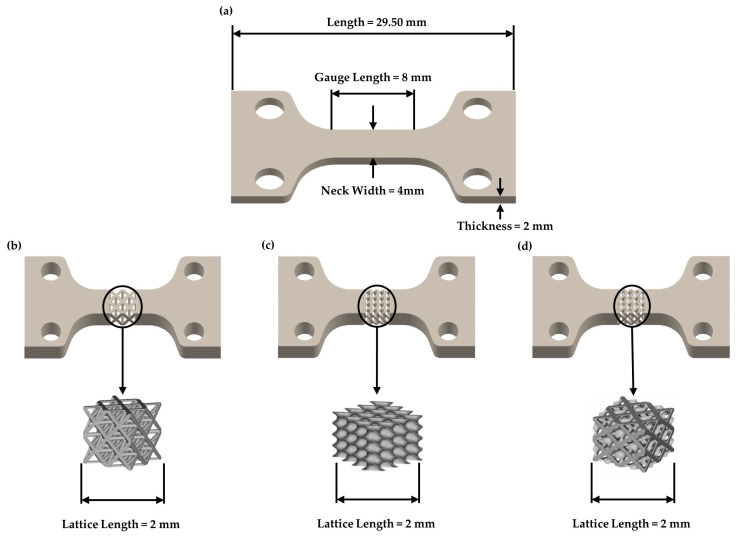
(**a**) Tensile dog bone specimens’ dimensions: (**b**) Octet lattice structure, (**c**) Schwarz Diamond (SD), (**d**) Hybrid (SD + FCC).

In order to investigate the influence of the different lattice structures on the mechanical behavior of Inconel 718 and their effect on the deformation mechanisms, dog-bone-shaped specimens were designed according to ISO 6892-1 standard with the aid of SolidWorks™ from Dassault Systemes SE (Vélizy-Villacoublay, France) software [15,33]. One strut-based (Octet), one TPMS (Schwarz Diamond), and one hybrid (SD-FCC) structure were applied to the effective area of the specimens [34]. The selected structures were designed as cubic unit cells with the aid of nTopology™ Version 5.4.2 from nTopology Inc. (New York, NY, USA) software, at three different relative densities (20–30–40% Octet., 30–40–50% SD, 40–45–50% SD&FCC). The relative densities were selected according to the discretization of each lattice structure; for example, it was not possible to achieve densities less than 40% in the SD&FCC structure. In Table 1, an overview of the designed specimens’ main characteristics such as the type of the applied lattice structure, the relative density, the length of the unit cell, and the thickness of the solid individual elements of the lattices known as strut thickness for the Octet structure and wall thickness for the TPMS-based structures. In Figure 2a, a digital illustration of the 3D printed solid dog bone specimen is presented, whereas the unit cells of the three different lattice structures that were applied to the specimen’s effective area is shown in Figure 2b–d. 

**Figure 3:**
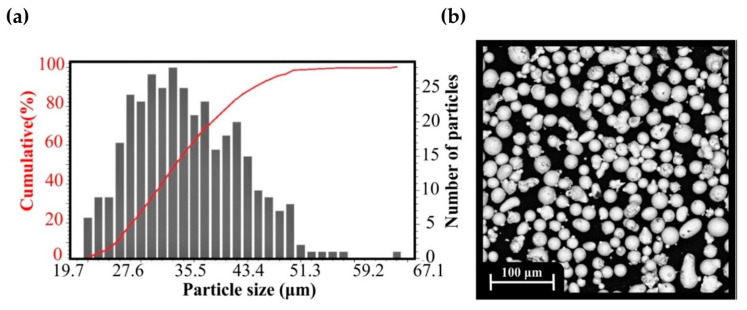
(**a**) Particle size distribution analysis; (**b**) SEM image of the powder particles [15].

The feedstock material used for the fabrication of the specimens was Inconel 718, a nickel-based superalloy in powder form from OC Oerlikon (Schwyz, Freienbach, Switzerland). Assessing the powder characteristics is vital for the application of the appropriate 3D printing conditions to achieve optimal printing quality results [35]. The powder characterization has already been conducted by previous research [15] to examine the powder morphological characteristics. Besides morphology, particle size distribution (PSD), the chemical composition of Inconel 718 was assessed. The evaluation of the chemical composition was carried out by Energy-Dispersive X-ray Spectroscopy (EDX) analysis as shown in Table 2. The particle size distribution along with a presentation of the powder microstructure as extracted by the Scanning Electron Microscopy are presented in Figure 3.

From previous studies [16,23], it is known that architected materials follow a specific behavior called scaling law; the difference between each scaling law reflects on whether a material is brittle or ductile and follows stretching-dominated or bending-dominated behavior. Materials with stretching-dominated behavior have high connectivity between their bonds, making the structure self-stressed and preventing bending phenomena, while on materials with low connectivity the bending phenomena are allowed. This behavior is dependent on the different types of lattice geometries. The relative density influences the mechanical properties through the size effect. For each architected material, this behavior tends to follow a specific curve. Mathematically, the effective properties and the overall behavior of each different lattice structure is described by the equation:

(1) $$\frac{\Phi_{lattice}}{\Phi_{solid}}=c\cdot {\left(p_{relative}\right)}^{n}$$

where *Φ* is the equivalent property (e.g., the Young’s Modulus E), *p* is the relative density of the lattice, and the variables *c* and *n* are constants of the equation. Their values vary depending on the behavior of the architected material. The *n* constant is an indicator of the stretching- or bending-dominated behavior of the structure. An *n* value close to 1 indicates a stretching-dominated behavior which means higher elastic and plastic deformations.

### 2.2. The 3D Printing of the Lattice Structure Specimens 

For the manufacturing of the specimens, the Selective Laser Melting (SLM) technique was utilized with the aid of ORLAS CREATOR from Coherent Inc. (Santa Clara, CA, USA) metal 3D printer. ORLAS CREATOR utilizes a continuous Yb-fiber laser beam with 250 W maximum power coupled with a wavelength of 1067 nm to successfully melt the metal powder. Furthermore, the maximum printing accuracy of the applied metal 3D printer reaches up to 25 μm at the vertical direction (layer height), and the minimum hatching distance was applied with a value of 40 μm. The 3D printing process was performed without the need of additional heating and the rotation of the scan vector was set to 45. The orientation was chosen according to the standards of ASTM 52921-13 [36]. The laser power was set at 160 W, and the scan speed was set at 1000 mm/s. For each type of tested structure and relative density, three identical dog bone specimens were manufactured. Volumetric Energy Density (VED) is strictly related to the printing quality with higher VED leading to lower scan speed and better dimensional accuracy [37,38]. The calculation of the VED is extracted by the following equation:

(2) $$VED=\frac{P}{v\cdot h\cdot t}\;\cdot 10^{6}\;J/mm^{3}$$

**Figure 4:**
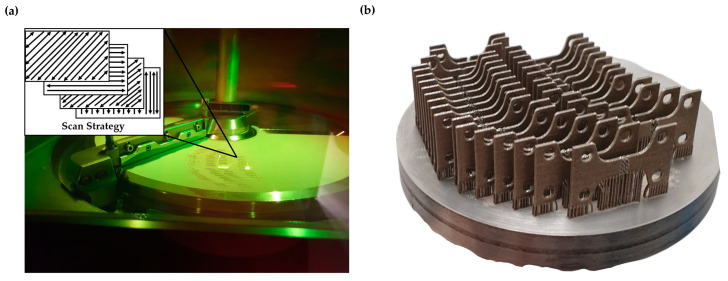
Illustration of the 3D printing process: (**a**) printing chamber and printing strategy; (**b**) final specimens as printed.

In this equation, P represents the laser power (W), v denotes the scan speed (mm/s), h is the hatching distance (μm), and t indicates the layer thickness (μm). In Figure 4, an overview of the 3D printing process is provided. The illustration includes the printing chamber during the printing process, the scan strategy employed, and the plate with the 3D printed specimens.

### 2.3. Microtensile Testing

**Figure 5:**
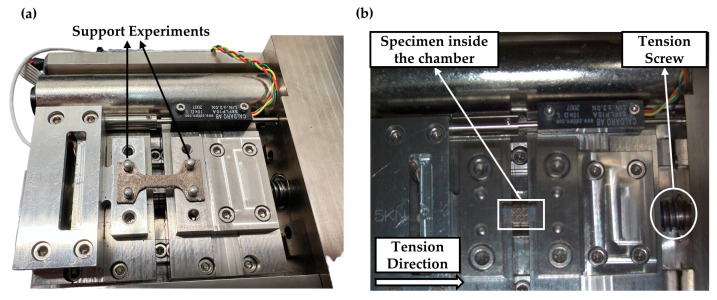
Deben Microtest MT5000 device: (**a**) specimen’s mounting on the micromechanical testing device; (**b**) specimen’s placement inside the SEM InTouch Scope JSM-IT510 chamber during the micro-tensile testing.

The fabricated specimens underwent in situ uniaxial tensile testing during SEM with the aid of Deben Microtest MT5000 from Deben Ltd. (Woolpit, UK) at room temperature and under vacuum according to ASTM E8 [27]. The maximum detected force was set to 5000 N, the motor speed was set to 0.2 mm/min, and the sampling rate at 100 milliseconds. The effective area was 4 mm^2^ for the bulk solid specimens, 1.5 mm^2^ for the solid specimens with notches, and 8 mm^2^ for the specimens with the applied lattice structures. The observation of the tensile testing and the failure mechanism was performed with the aid of SEM InTouch Scope JSM-IT510 from Jeol Ltd. (Tokyo, Canto, Japan). Also, an elemental analysis of the specimens was conducted with the aid of the energy-dispersive (EDS) X-ray detector DrySD™ Detector Unit that utilizes a silicon drift detector (SDD) for the SEM, which gave the equivalent of the chemical composition characterization of the used Inconel 718 powder. The electron gun beam current is measured with the aid of the MP-90090PCD Probe Current Detector installed inside the SEM, and the movement of the electron gun providing the field of view to the observer is made with the aid of the MP-95030TB trackball allowing the movement on X, Y, and Z axes by stage control. During all the experiments, the chamber’s height was set at 30 mm and the electron gun was set at 106 μA [39]. An image of the experimental setup is portrayed in Figure 5a at a zero elongation and in Figure 5b during the tensile process.

### 2.4. Finite Element Analysis

**Figure 6:**
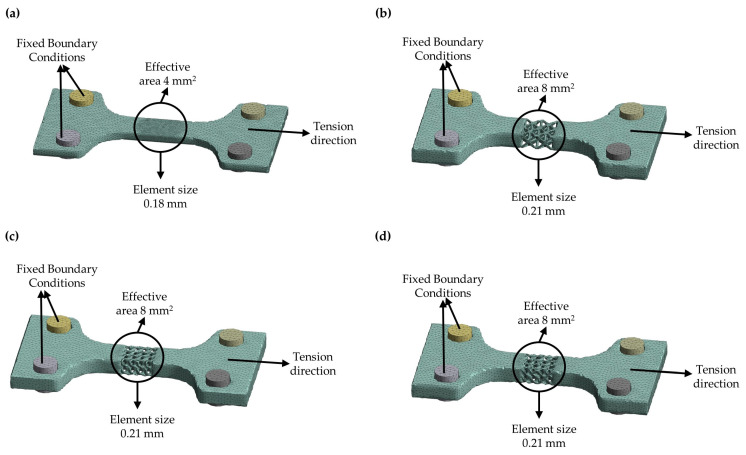
Specimens after the mesh generation phase: (**a**) Solid Specimens; (**b**) Octet Specimens; (**c**) Schwarz Diamond (SD) Specimens; (**d**) Schwarz Diamond and Face Centered Cubic (SD&FCC) specimens.

The mechanical behavior of the 3D designed specimens was also investigated with the Finite Element Analysis (FEA) method, utilizing the ANSYS™ software from ANSYS, Inc. (Canonsburg, PA, USA). Regarding the boundary conditions, the left gripped end of the specimen shown in Figure 5a was considered fixed, while at the right gripped end an appropriate velocity was applied to achieve the equivalent experimental deformation after the analysis. A bilinear isotropic strain hardening model was selected for the feedstock material based on the experimental results. The elastic modulus was set at 80700 MPa, the yield strength was set at 811 MPa, and the tangent modulus of the hardening region was set at 3000 MPa till the breaking point of 1010 MPa. The explicit dynamic module was utilized to capture the mechanical behavior of the different lattice structures. The mesh sensitivity analysis by utilizing convergence studies was performed in order to reach mesh-independent results normalizing the equivalent von Mises stresses. The convergence studies showed that adequate mesh-independent results occurred with the use of around 125,000 elements, with finer mesh around the effective area of the specimen. The element size ranged between 0.2 and 0.8 mm, with the smaller elements surrounding the effective area where the rupture was expected. Figure 6 shows the results of the mesh generation for the specimens, while Table 3 provides the element size range and total number of elements for each type of specimen. 

## 3. Results and Discussion

### 3.1. Mechanical Charaterization Assisted with FE Analysis

**Figure 7:**
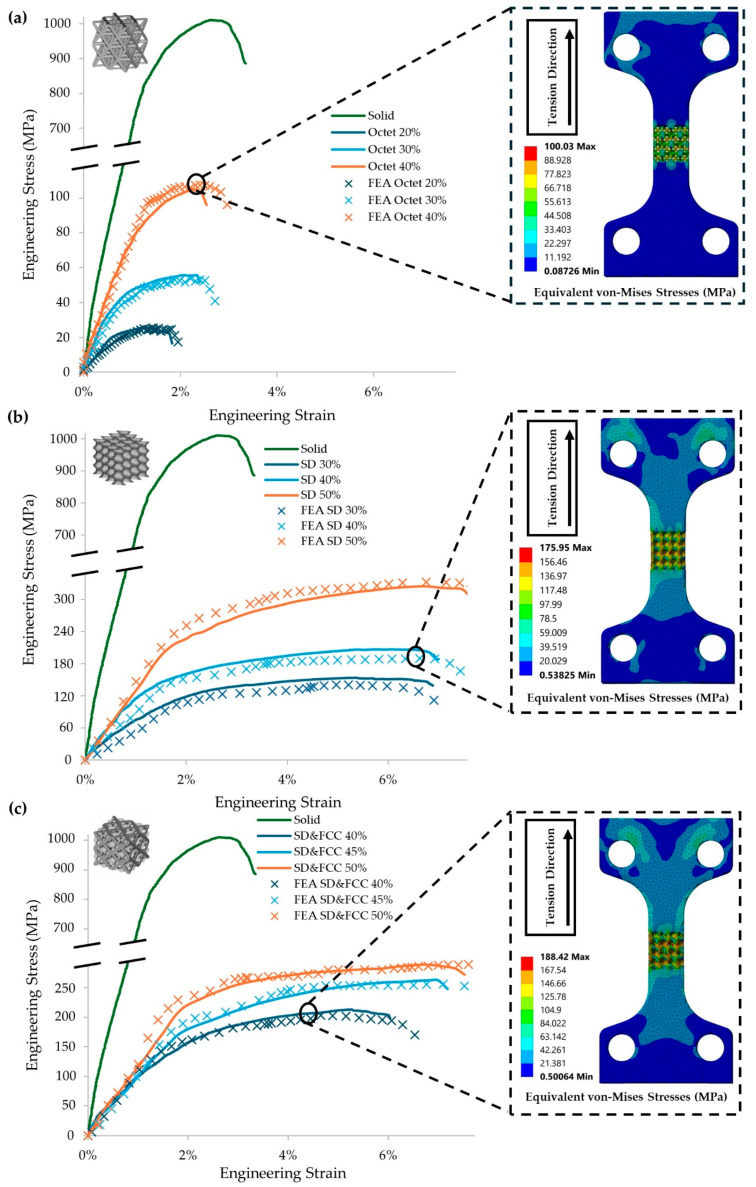
Stress–strain response of (**a**) Octet, (**b**) Schwarz Diamond, and (**c**) Schwarz Diamond and Face Centered Cubic hybrid structure, along with the FEA-generated curves and the stress distribution on the tensile specimens.

To assess the mechanical response of the different lattice geometries, all the specimens went under quasi-static uniaxial tensile testing, identifying the main bulk properties such as the modulus of elasticity (Young’s Modulus, *E*), the yield strength (*σ_y_*), and the ultimate tensile strength (*UTS*) of Inconel 718 as a reference point and the effect of the different lattice geometries on the mechanical strength of the structure compared to the conventional bulk structure. With the support of Finite Element Analysis (FEA), a simulation of the uniaxial testing was conducted, verifying the reliability of the experimental results and allowing the identification of scaling laws regarding the effect of the different relative densities of the applied lattice geometries on the mechanical performance of the final structure. In Figure 7, the mechanical response of each type of specimen, (a) Octet, (b) Schwarz Diamond (SD), (c) hybrid Schwarz Diamond, and FCC (SD&FCC), is illustrated by the equivalent engineering stress–strain curve, comparing them with the solid specimens’ response and with the results of the FEA analysis along with the contours for the equivalent von Mises stresses for each type of architected material at a relative density of 40%.

As it is observed in Figure 7a–c, applying a lattice geometry to the effective area results in reduced maximum strength, as is observed from the peak points of the equivalent curves. The reduction percentage is relevant to the relative density on the effective area compared to the equivalent volume in the solid state. In this case, a 60% reduction in the material volume resulted in an 80% reduction in the tensile strength compared to the conventional one. This drop is expected, and it is relevant to the type of lattice and its relative density. Topology optimization leads to structures with the optimal strength to weight ratio and can lead to structures with tailored mechanical properties for specialized applications. Besides the differences in the overall strength of the final structure, it was also observed that the TPMS (Schwarz Diamond, SD) and the TPMS-based (SD + FCC) hybrid lattice structures present higher engineering strains than the strut and even the solid structure. Also, it is clear that the TPMS and the hybrid lattices present higher engineering performance compared to the strut-based lattices (Octet) regarding the strength and elasticity of the structures, a conclusion that has been verified from the results of Finite Element Analysis and the results of previous studies [23,34]. Due to the rigidity of the metal, this increase in the engineering strain results in a wider plastic deformation area, resulting in higher energy absorption for the TPMS-based structures. The increased ductility of the TPMS-based lattices (SD and SD&FCC) is also visible due to the necking on the gauge length of the specimens in the contours of the equivalent von Mises stresses that are presented in Figure 6c. It is apparent that the maximum stresses are present in the center of the gauge length area; also, some mild stresses seem to appear in the gauge length area and the area surrounding the moving gripped end where the tensile force t is applied. In Table 4, the overall results regarding the mechanical properties of the 3D printed specimens are presented by illustrating the key factors of ultimate tensile strength (UTS), the yield strength, the Young’s Modulus of elasticity (E), and the elongation at break.

### 3.2. Identification of the Scaling Laws of Different Lattice Structures

**Figure 8:**
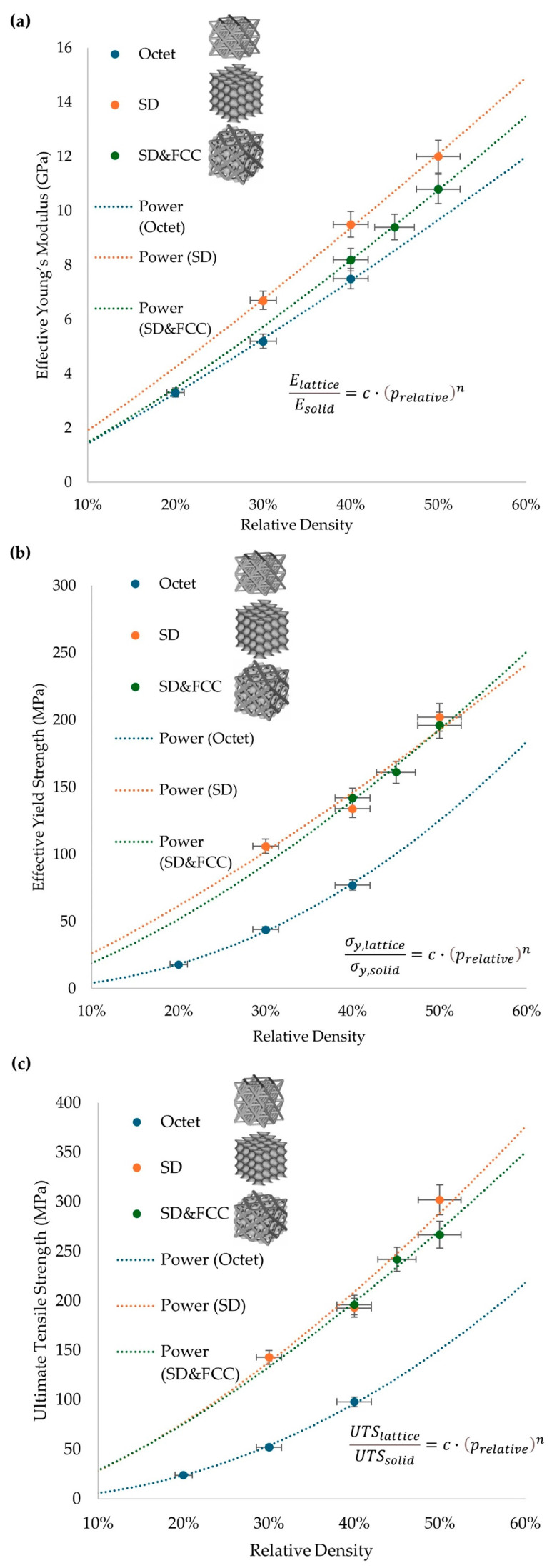
Quantitative diagrams for each lattice structure: (**a**) effective Young’s Modulus, (**b**) effective yield strength, (**c**) ultimate tensile strength.

The overall results from Table 4 reveal a distinct type of behavior for each lattice structure. It is also obvious that the relative density of the architected material reflects on the final mechanical properties of the overall structure. The curves are extracted by the experimental results that are stated in Table 4 and with the utilization of Equation 1 for the equivalent property, an identification of the main constants that describe the scaling laws of lattice structure (Octet, SD, SD&FCC) for each of the material’s main mechanical properties (Young’s Modulus (*E*), yield strength (*σ_y_*), ultimate tensile strength (*UTS*)), as presented in Figure 8.

In Figure 8a, it is observed that all the applied lattice structures present an overall mild stretching-dominated behavior. This is visible in the curvature, which is approaching linear behavior. In Figure 8b,c, it is visible by the curvature that the SD and SD&FCC structures have an almost linear behavior regarding the influence of the relative density on the mechanical performance. On the other hand, the Octet structure presents very low strength at low densities and significantly higher strength at higher densities, with the curvature in this situation approaching exponential behavior. In Table 5, the final values of the constants c and *n* for the Young’s Modulus (*E*), yield strength (*σ_y_*), and ultimate tensile strength (*UTS*) are presented for the experimental results for each architected material. The adequate c-value range is between 0 and 4, and the projected *n*-value is between 1 and 2.5.

### 3.3. Identification of the Deformation Mechanisms

**Figure 9:**
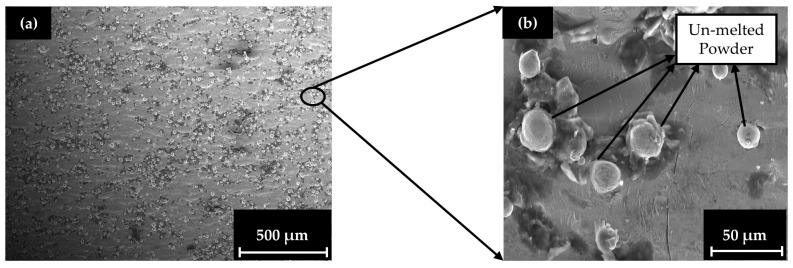
Micrographs of the 3D printed specimens’ surface: (**a**) low-magnification image of the specimens’ surface, (**b**) high-magnification image of an area with significant defects.

In order to assess the overall quality of the printing process, an examination of the specimens’ microstructure was conducted with SEM analysis utilizing a high-energy Backscattered Electrons (BSEs) detector and a low-energy Secondary Electrons (SEs) detector for the capture of the surface morphology. In Figure 9, a demonstration of the specimens’ surface and structural characteristics after the printing process before the mechanical testing is presented with the aid of SEM. The illustration includes a general view of the printed specimen’s surface (Figure 9a) and a focus on the possible printing defects of the final structure (un-melted particles, contaminated particles) (Figure 9b).

**Figure 10:**
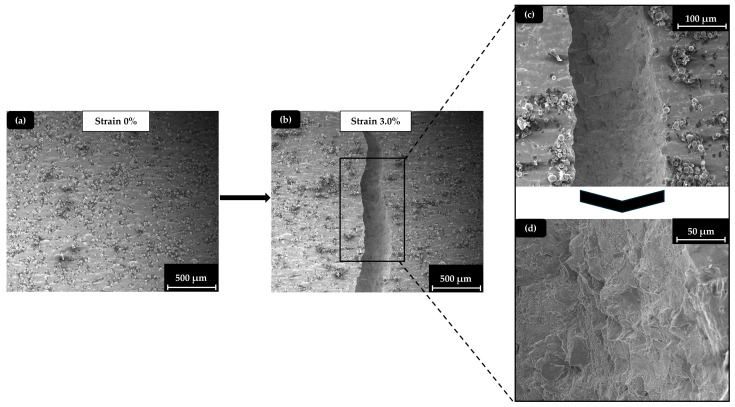
SEM images of a solid specimen tested: (**a**) at the initial phase (strain 0%); (**b**) at the end phase (strain 3.0%); (**c**) fractured surface; (**d**) detail inside the crack area.

Although the printing conditions are described as optimal, there are still visible instances of un-melted powder particles and powder contamination. In general, high percentages of un-melted powder particles could lead to areas with significant voids due to non-sintered successive particles, leading to reduced mechanical properties. It is clearly visible in the low-magnification image of the specimen’s surface that such phenomena still exist. In the high-magnification image, though, after focusing on the areas that had a significant number of dots (un-melted particles), it is obvious that the overall percentage of the un-melted powder is relatively low, and the un-melted particles are sparse and not in direct contact with each other, resulting in minimal defects. In Figure 10, a demonstration of the solid specimen’s surface during the microtesting process is made. The illustration includes an overview of the solid specimen at the beginning of the experiment (Figure 10a), an overview of the solid specimen after the end of the experiment (Figure 10b), and a demonstration of the essential details that describe the fracture mechanism on the surface (Figure 10c) and inside the crack area (Figure 10d).

**Figure 11:**
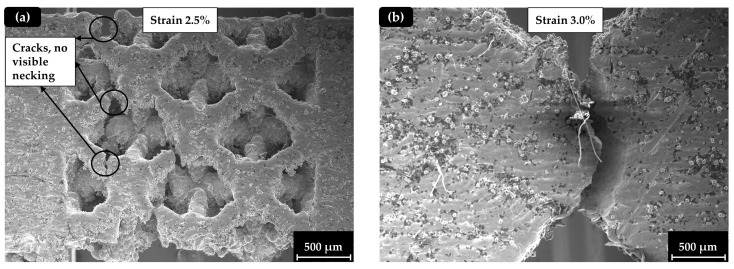
Fractography analysis: (**a**) Octet lattice structure (40%); (**b**) solid structure with a notch.

As it is observed in Figure 10, the solid specimen presented a brittle behavior during the crack initiation, highlighted by the vertical crack formation and the absence of the necking phenomenon that signals significant plastic deformation. This is also confirmed by the relatively low final value of strain at the breaking point. Specimens with a strut (Octet) lattice structure presented significantly lower strength than TPMS and hybrid specimens, but this fragility did not result in ductility change. In Figure 11a,b, the strut (Octet) structure and the solid with a notch specimen, respectively, are presented after the experiment, confirming the above statement. It is worth noting that, although the Octet specimens exhibited lower strength and a smaller plastic deformation area compared to the TPMS-based structures, they also displayed a unique deformation mechanism, with more discrete fracturing of individual lattices. In contrast, the TPMS-based structures showed a deformation mechanism that more closely resembled the continuous fracture observed in solid specimens.

**Figure 12:**
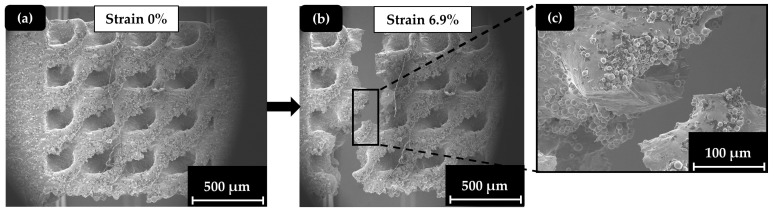
SEM images for Schwarz Diamond (SD) specimens (40%): (**a**) initial phase (strain 0%); (**b**) end phase (strain 6.9%); (**c**) deformation detail at the surface.

In contrast, the TPMS and hybrid structures presented higher deformations and visible necking during the micro-tensile tests, confirming the results of Figure 7. In Figure 12, a demonstration of the Schwarz Diamond specimen’s surface during the testing process is made. The illustration includes an overview of the Schwarz Diamond specimen at the beginning of the experiment (Figure 12a), an overview of the Schwarz Diamond specimen after the end of the experiment (Figure 12b), and a demonstration of the essential details that describe the crack mechanism on the surface (Figure 12c). 

**Figure 13:**
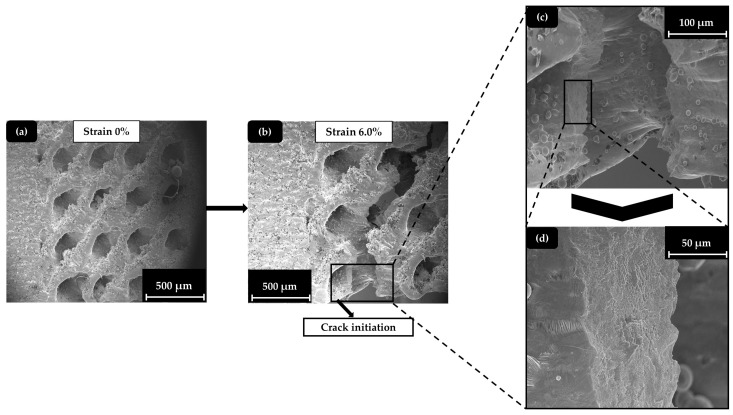
SEM images for hybrid (SD&FCC) specimens (40%): (**a**) initial phase (strain 0%); (**b**) end phase (strain 6.0%); (**c**) deformation detail at surface; (**d**) deformation detail inside the crack.

In Figure 13, a demonstration of the hybrid (SD&FCC) specimen’s surface during the testing process is made. The illustration includes an overview of the hybrid (SD&FCC) specimen at the beginning of the experiment (Figure 13a), an overview of the hybrid (SD&FCC) specimen after the end of the experiment (Figure 13b), and a demonstration of the essential details that describe the crack mechanism on the surface (Figure 13c) and inside the crack area (Figure 13d).

**Figure 14:**
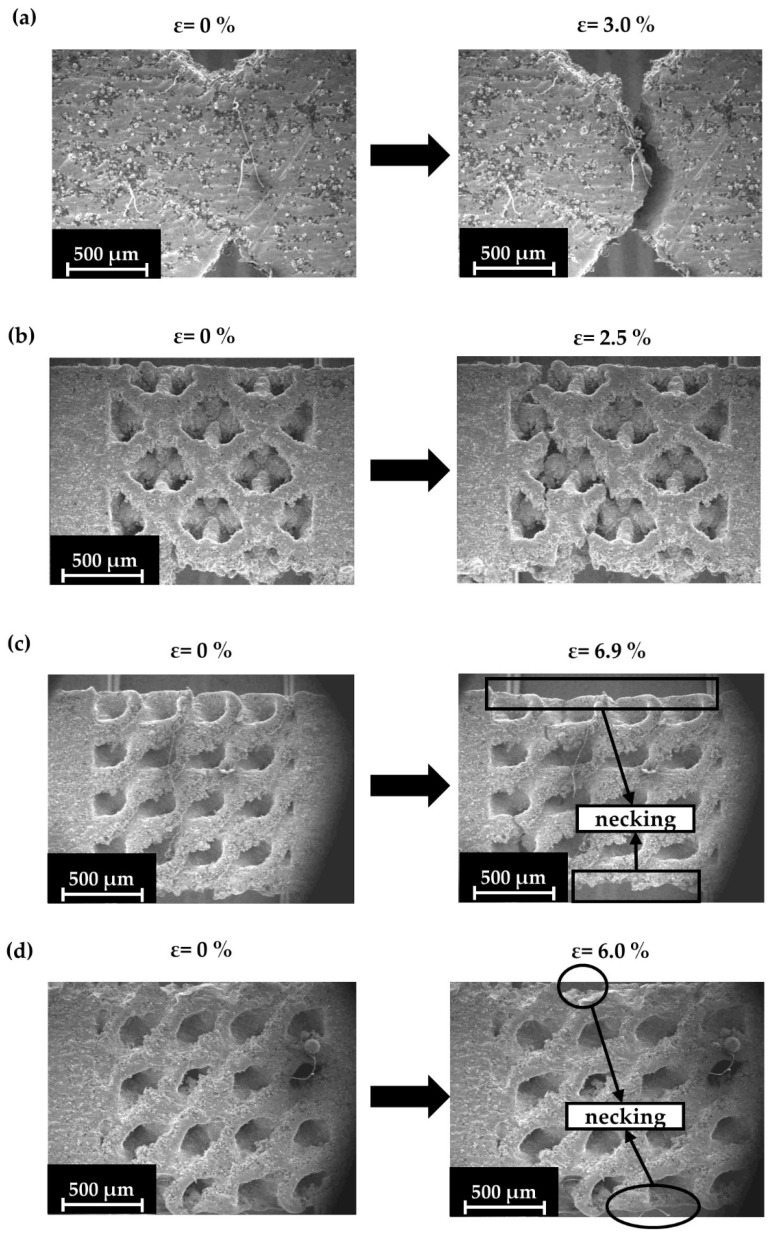
SEM images for all specimens at the beginning of the experiment and at the moment of the first crack: (**a**) Solid; (**b**) Octet; (**c**) Schwarz Diamond; (**d**) Hybrid Schwarz Diamond and Face Centered Cubic.

The experiments revealed that the necking of the Schwarz Diamond and the Schwarz Diamond and Face Centered Cubic hybrid specimens was more pronounced compared to the Octet and solid specimens, indicating a significantly larger plastic deformation region. Also, the more pronounced necking indicates an extended plastic region and a stretching-dominated behavior, in contrast to the brittle behavior of solid materials. In Figure 14, images of each specimen type are shown, depicting the start of the experiment and the moment just before crack detection.

It is expected that the Schwarz Diamond specimens present significantly higher ductility, which is something that is confirmed in the results of Figure 7. This is also visible from the necking that is portrayed in Figure 14c. The same phenomena during the testing were observed in the responses of the hybrid (Schwarz Diamond and Face Centered Cubic) specimens (Figure 14d). It was observed that Schwarz Diamond specimens presented the highest ductility of all types of specimens while also maintaining better overall strength (UTS) compared to the other types of lattice structures. In contrast, Octet and Solid specimens presented similar behavior, with minimal plastic deformation region and brittle deformation mechanisms. The results from the SEM experiments confirm the experimental and FEA curves that are presented in Figure 7 describing the behavior of the different types of lattice geometries.

## 4. Conclusions

The objective of this research was the evaluation of the micro-tensile response of strut-based and TPMS-based lattice geometries and the effect of the relative density on the mechanical properties of the final structure for the production of Inconel 718 superalloy advanced architected materials. The application of different lattice geometries led to structures with higher stretching-dominated behavior compared to the solid structure, leading to improved ductility resulting in higher energy absorption, making them suitable for applications that require a good impact response, especially in the case of the SD and the SD&FCC hybrid structures. In both structures, at 50% relative density, an increase in the plastic deformation range was observed compared to the solid and the strut structure (7.5% elongation at break compared to the solid’s 3.0%). The SD structure showed slightly better toughness in higher stresses compared to the SD&FCC at the breaking point (UTS of SD 50% 303 MPa, UTS of SD&FCC 50% 267 MPa), but both of these structures presented similar mild stretching-dominated behavior as was observed from the scaling laws (Young’s Modulus *n* for SD = 1.14, for SD&FCC = 1.23), and had an almost linear relationship between the relative density and the mechanical response (yield strength *n* for SD = 1.24, for SD&FCC = 1.44). In contrast, the Octet structure presented lower toughness (UTS of Octet 40%, 98 MPa) at the breaking point, and the dependence of the relative density on the mechanical response was significantly higher and approached an exponential relationship (yield strength *n* for Octet = 2.10). From the study of the microstructure, although all the different lattice structures were characterized by stretching-dominated behavior, the Octet tolerated fewer strains, leading to a relatively small plastic region compared to the SD and the SD&FCC structures, which were characterized by brittle behavior. The SD and SD&FCC structures had better overall energy absorption, as it was observed by the area on the stress–strain curve, while simultaneously having less than half the density of the conventional solid structure, making them suitable candidates for applications that require high impact resistance, such as protective equipment (armor, helmets). Furthermore, the optimal weight reduction of 50–60% (r.d. 40–50%) led to a 70–75% reduction in the overall strength of the final structure (UTS solid structure 1010 MPa, UTS of SD 50% 303 MPa). The maintenance of the strength to weight ratio, combined with the improvement of the energy absorption can lead to the production of lightweight parts with high crashworthiness that are adequate for automotive (spoilers, bumpers) or aerospace (turbine blades) applications.

## Figures and Tables

**Table 1:** Overview of the tested specimens.

Feedstock Material	Specimens’ Geometry	Relative Density	Unit Cell Length (mm)	Strut/Wall Thickness (mm)
Inconel 718	Octet	20%	2	0.274
		30%		0.347
		40%		0.414
	Schwarz Diamond (SD)	30%	2	0.258
		40%		0.344
		50%		0.428
	Schwarz Diamond and Face Centered Cubic (SD&FCC)	40%	2	SD: 0.291 FCC: 0.291
		45%		SD: 0.325 FCC: 0.325
		50%		SD: 0.360 FCC: 0.360

**Table 2:** Chemical composition as extracted by EDX analysis [15].

Chemical Element	Chemical Composition (wt.%)
Nickel (Ni)	54.11
Chromium (Cr)	18.14
Iron (Fe)	17.93
Niobium (Nb)	4.16
Molybdenum (Mo)	2.95
Titanium (Ti)	1.12
Other	1.59

**Table 3:** Finite Element Analysis mesh generation properties.

Specimens’ Geometry	Number of Elements	Element Size Range (mm)
Solid	125,871	0.18–0.80
Octet	126,724	0.21–0.60
SD	127,020	0.21–0.60
SD&FCC	126,929	0.21–0.60

**Table 4:** Mechanical properties of the 3D printed specimens.

Specimen’s Type	E (GPa)	Yield Strength (MPa)	UTS (MPa)	Elongation at Break (%)
Solid	80.7 ± 7.3	811 ± 34	1010 ± 70	3.0 ± 0.4
Octet 20%	3.3 ± 0.5	19 ± 3	24 ± 5	1.5 ± 0.2
Octet 30%	5.2 ± 0.4	44 ± 4	52 ± 9	2.4 ± 0.3
Octet 40%	7.5 ± 0.4	77 ± 3	98 ± 5	2.5 ± 0.2
SD 30%	6.7 ± 0.6	106 ± 5	143 ± 6	6.8 ± 0.3
SD 40%	9.5 ± 0.7	134 ± 8	193 ± 8	6.9 ± 0.6
SD 50%	12.0 ± 1.1	202 ± 11	303 ± 16	7.5 ± 0.8
SD&FCC 40%	8.2 ± 0.6	142 ± 9	196 ± 13	6.0 ± 0.7
SD&FCC 45%	9.4 ± 0.5	161 ± 9	242 ± 15	6.8 ± 0.8
SD&FCC 50%	10.8 ± 1.4	199 ± 12	267 ± 19	7.5 ± 0.9

**Table 5:** Scaling laws constants for each architected material.

Lattice Structures	Young’s Modulus (*E*)		Yield Strength (*σ_y_*)		Ultimate Tensile Strength (*UTS*)	
	c	*n*	c	*n*	c	*n*
Octet	0.27	1.18	0.67	2.10	0.61	2.02
SD	0.33	1.14	0.56	1.24	0.78	1.44
SD&FCC	0.31	1.23	0.65	1.44	0.71	1.39

## Data Availability

Data sharing is not applicable.
